# The goal of blood pressure in the hypertensive patient with diabetes is defined: now the challenge is go from recommendations to practice

**DOI:** 10.1186/1758-5996-6-31

**Published:** 2014-03-04

**Authors:** Patricio Lopez-Jaramillo, Jose Lopez-Lopez, Cristina Lopez-Lopez, Miguel I Rodriguez-Alvarez

**Affiliations:** 1 Research Institute and Clinic of Diabetes and Metabolic Syndrome, Fundación Oftalmológica de Santander (FOSCAL), Calle 155 A No 23-09, Torre Milton Salazar, Urbanizacion El Bosque, Floridablanca, Santander, Colombia; 2 MASIRA Research Institute, Medical School, Universidad de Santander (UDES), Bucaramanga, Colombia; 3 Medical School, Universidad Autonoma de Bucaramanga (UNAB), Bucaramanga, Colombia

**Keywords:** Hypertension, Diabetes, Guidelines, Blood pressure

## Abstract

The recent Latin American and European guidelines published this year has proposed as a goal for blood pressure control in patients with diabetes type 2 a value similar or inferior to 140/90 mmHg. High blood pressure is the leading cause of cardiovascular diseases and deaths globally. Although once hypertension is detected, 80% of individuals are on a pharmacologic therapy only a minority is controlled. Diabetes also is a risk factor for other serious chronic diseases, including cardiovascular disease. Whether specifically targeting lower fasting glucose levels can reduce cardiovascular outcomes remains unknown. Hypertension is present in 20% to 60% of patients with type 2 diabetes, depending on age, ethnicity, obesity, and the presence of micro or macro albuminuria. High blood pressure substantially increases the risk of both macro and micro vascular complications, doubling the risk of all-cause mortality and stroke, tripling the risk of coronary heart disease and significantly hastening the progression of diabetic nephropathy, retinopathy, and neuropathy. Thus, blood pressure lowering is a major priority in preventing cardiovascular and renal events in patients with diabetes and hypertension. During many years the BP goals recommended in patients with diabetes were more aggressive than in patients without diabetes. As reviewed in this article many clinical trials have demonstrated not only the lack of benefits of lowering the BP below 130/80 mmHg, but also the J-shaped relationship in DM patients. Overall we discuss the importance of define the group of patients in whom significant BP reduction could be particularly dangerous and, on the other hand, those with a high risk of stroke who could benefit most from an intensive hypotensive therapy. In any case, the big challenge now is avoid the therapeutic inertia (leaving diabetic patients with BP values of 140/90 mmHg or higher) at all costs, as this would lead to an unacceptable toll in terms of human lives, suffering, and socioeconomic costs.

## Introduction

The recent Latin American [1] and European [2] guidelines published this year has proposed as a goal for the blood pressure control in patients with diabetes type 2 a value similar or inferior to 140/90 mmHg. The American Diabetes Association (ADA) [3] guidelines recommended similar value for systolic blood pressures but for diastolic blood pressure the recommended value was lower that 80 mmmHg.

The important role that increased levels of blood pressure have as one of the principal risk factors to myocardial infarction (MI) [4] and stroke [5], made this mater one of special interest that need be very well supported and universally accepted in perspective of improve the lowers levels of hypertensive control reported worldwide. Recently, the Prospective Urban Rural Epidemiology (PURE) study demonstrated the high prevalence of hypertension and the very low awareness, treatment and control of hypertension worldwide [6]. This community based study included 153,996 adults (35–70 years) from 628 rural and urban communities from three high- income countries (HICs), 10 upper middle and low middle income (UMIC and LMIC) and four low-income countries (LIC) in various parts of the world. Hypertension was defined when individuals reported treatment for hypertension or had an average blood pressure (BP) greater than 140/90 mmHg from two measures of resting sitting BP using an automated digital device. Overall, 40.7% of participants were found to have hypertension, with 13.3% having a BP of at least 160/100 mmHg and 4.4% a BP of at least 180/110 mmHg. Of those with hypertension, 46.4% were aware of this condition, 40.6% were on pharmacological treatment, but only 13.1% had BP controlled (<140/90 mmHg). Overall, 12.5% of treated hypertensive patients received two or more BP lowering medications, with a decreasing trend from wealthier to poorer countries (HIC, 18.1%, UMIC 14.5%, LMIC 14.1%, LIC 1.6%; P < 0.0001). Hypertension prevalence was highest in participants with diabetes (63%), and even though awareness was 74.4%, and the percentage of those who received treatment 69.3%, the control rate was only 23.3%. So, it is crucial to improve the control of blood pressure in a group of high risk, as is the diabetic population.

In the present article we review the crucial role of hypertension and diabetes in the risk of develop cardiovascular diseases (CVD) and the evidences that support the decision of the Latin Americans and Europeans experts, with the aim of motivating to the health team and the subjects affected of hypertension and diabetes to participate actively in the challenge to pass from the guidelines recommendations to the clinical practice and public health programs to improve the percentage of control of blood pressure.

### The role of hypertension in the global burden of cardiovascular disease

Cardiovascular diseases are the major causes of mortality and morbidity globally and affect over 50% of men and 40% of women over their lifetimes [7,8]. Although age adjusted mortality for CVD is decreasing in developed countries, there is a sharp rise in developing countries [9,10]. In 1990, 5.3 million CVD deaths occurred in developed countries, whereas there were about 9 million deaths from CVD in developing countries [11]. In addition, CVD in developing countries occurs at a younger age. In the same year (1990) the proportion of deaths due to CVD in those <70 years in developed countries was 26.5% of total deaths, while in developing countries it was 46.7%. It was estimated that the disability adjusted life years (DALY’s) lost in developing countries was about three times greater than that in the developed countries [12]. Consequently, approximately three quarters of the global mortality and perhaps about 80% of the disease burden (measured as DALY’s lost) is expected to occur in low and middle income countries (L + MIC) by the year 2020 [13]. These numbers have not materially changed in the last years; Thus, in 2011 two of 3 deaths each year are attributable to non-communicable diseases (mainly heart disease, stroke, diabetes, cancers and chronic respiratory disease) with four fifths of the deaths occurring in L + MIC, with a third occurring in people younger than 60 years. Overall, age specific non- communicable disease deaths are two times higher in L + MIC than in high-income countries [14-16].

High blood pressure BP is the leading cause of CVD and deaths globally. It is associated with 7.5 million deaths (which represents one-eighth of all deaths) per year worldwide [7,8]. The importance of BP as a modifiable risk factor for CVD is well recognized and many effective and inexpensive BP-lowering treatments are commonly available [17,18]. Therefore, BP control and prevention of related morbidity and mortality could be clearly achievable. However, the awareness, treatment and control of hypertension are low in all countries, despite hypertension being an easily detectable, common and well established risk factor for CVD. The reasons for a persisting huge gap in awareness and treatment of hypertension, despite the identification and control of blood pressure being prioritized by many national and global organizations and despite the availability of cheap and effective medications are unclear. Use of combination therapies, which is needed to control hypertension, is relatively uncommon even in wealthier countries. Although once hypertension is detected, 80% of individuals are on a pharmacologic therapy only a minority is controlled [6]. This suggests the need for a more aggressive approach to BP control (e.g. by using combination therapies). A recent study [19] using a meticulously algorithm applied to DM2 patients was able to lower BP, however more than half of the patients did not achieve the ADA/Joint National Committee on Prevention, Detection, Evaluation, and Treatment of High Blood Pressure: The Seventh Report (JNC 7) targets demonstrating the complexity of BP control in this population.

The BP Lowering Treatment Trialists Collaboration (BPLTTC) meta-analysis of individual patient data from over 160,000 participants in 29 trials showed that lowering systolic BP by 5 mmHg over 4 to 5 years with most drugs, including blockers of the renin-angiotensin- aldosterone system (RAAS) reduced the risk of ischemic heart disease (IHD) by 20%, stroke by 28% and major CVD events by 22%, with additional reductions in heart failure [18]. Since five treatment groups (diuretics, beta blockers, angiotensin converting enzyme inhibitors, angiotensin receptors blockers and calcium channel blockers) have demonstrated not only anti-hypertensive but also cardio-preventive efficacy, as well as renal and cerebrovascular protection, all of them are of choice in patients with hypertension and diabetes type 2 (DM2). However, when selecting to start treatment with monotherapy, drugs that blocking the renin-angiotensin-aldosterone system should be primarily prescribed for its nefro-protective effect. Angiotensin receptors blockers (ARBs) are better tolerated, and this is very important issue in patients with chronic diseases such as hypertension and DM2 where adherence is essential. As a general rule, a long acting drugs providing protection for 24 hours must be indicated in order to use single doses, which offer greater protection and improve patient adherence to treatment [20,21]. Recently, a systematic review and bayesian network meta-analysis comparing the effectiveness of renin-angiotensin system blockers and other antihypertensive drugs in patients with diabetes, have concluded that the reno-protective effects and superiority of using ACE inhibitors in patients with DM2, and also that the available evidence is not able to show a better effect for ARBs compared with angiotensin converting enzyme inhibitors (ACEI). Calcium channel blockers might be the preferred treatment in combination with ACEI if adequate blood pressure control cannot be achieved by ACEI alone [22].

### Diabetes and cardiovascular diseases

Diabetes is a metabolic disorder that is diagnosed when the fasting and/or post load glucose level rises above well-established thresholds. For instance, the criteria for diagnosis of DM2 adopted and recommended by the Latin American Consensus [1], are listed as follows:

1. Fasting glucose at least 126 mg/dl in two successive readings

2. At least 200 mg/dl 120 min after oral glucose tolerance test

3. At least 200 mg/dl at any time in the presence of symptoms

These thresholds were chosen because they identified people at particularly high risk for retinopathy based on epidemiological data. These data also have shown that people with diabetes and poorly controlled glucose levels have higher risks of retinopathy than people with diabetes and well-controlled glucose levels [23,24]. Diabetes also is a risk factor for other serious chronic diseases, including cardiovascular disease. Indeed, a recent metaanalysis of large prospective studies comprising 450.000 people showed that men and women with diabetes are 2 and 3 times more likely, respectively, to die of coronary heart disease than men and women without diabetes [25]. Moreover, epidemiological analyses of prospective data from the United Kingdom Prospective Diabetes Study (UKPDS), which recruited people with newly diagnosed diabetes showed that progressively higher HbA1c levels predicted higher hazards of severe retinal or renal disease, cataracts, myocardial infarction, heart failure, amputation or peripheral vascular disease, stroke, and death [26]. They also showed that the risk relationship differed with respect to outcome, with a stronger relationship to some outcomes and a weaker relationship to others. Thus, organ systems may vary in their susceptibility to damage related to glucometabolic abnormalities, a proposal strongly supported by epidemiological analysis [27].

Whether specifically targeting lower fasting glucose levels can reduce cardiovascular outcomes remains unknown. Some clues may reside in the 10-year-long UKPDS, which randomized people with newly diagnosed diabetes and few other cardiovascular risk factors to a policy of targeting fasting plasma glucose 108 mg/dl versus a conventional policy targeting fasting plasma glucose 270 mg/dl [28,29]. This study reported a clear reduction in myocardial infarction and death in a subset with recently diagnosis of DM2 and obese participants allocated to metformin as the means of glucose lowering, with no significant cardiovascular effects in the other participants [29]. Moreover, after 9 years of passive follow-up, all participants experienced a 13% and 15% reduction in death and myocardial infarction, respectively, and the subset given metformin retained the benefit observed during the active treatment phase [30]. These results have support the proposal that the intensive therapy to normalize blood glucose in perspective of prevent CVD is effective only if is started from the very begging diagnosed of DM2 [31].

Individuals with DM2 are less likely to survive a first myocardial infarction than their no diabetic peers. Therefore, early identification of coronary artery disease (CAD) in the diabetic population is needed. The mechanism of acute coronary syndrome and their implications for therapy haven recently reviewed [32], included the role of hyperglycemia [33]. However, the fact that CAD is often asymptomatic in diabetic patients makes such identification a challenge. A number of studies have shown that silent myocardial ischemia as evidenced by non-invasive tests such as the electrocardiogram stress test, myocardial scintigraphy or stress echocardiography affects 20-50% of diabetic patients with additional risk factors [34,35]. The term of silent ischemia includes an entity named true silent myocardial ischemia or clandestine ischemia, which is characterized by myocardial perfusion defects in the absence of both angina and ST-segment depression > 1 mm during the exercise test. In the largest study performed to evaluate the prevalence of silent myocardial ischemia in diabetic patients, the DIAD (Detection of Ischaemia in Asymptomatic Diabetics) study [34] reported that patients with DM2 have a high prevalence of silent myocardial ischemia and true silent myocardial ischemia. The detection of asymptomatic CAD was associated with a higher number of interventions, but without a benefit in outcomes. Recently [35] it was evaluate the prevalence of true silent myocardial ischemia in asymptomatic DM2 patients in comparison with a non-diabetic control group. Risk factors of CVD were similar between both groups. The prevalence of true silent myocardial ischemia was strikingly higher in DM2 than in their no diabetic peers (21.9% vs. 2.4%). As previously reported in silent myocardial ischemia, men are more likely to have perfusion defects. Moreover, as occurs in silent ischemia the number of traditional risk factors is not useful as a means of identifying patients with true silent myocardial ischemia [36]. An important finding was that diabetes retinopathy (DR) is independently associated with the presence of true silent myocardial ischemia. DR has been recently recognized as an indicator of risk for CAD in diabetic patients [36] and it is predictive of cardiovascular mortality [37]. A recent meta-analysis that have included 20 studies and 19,234 subjects have shown that DR predicts all-cause mortality and CV events in both type 1 and 2 diabetic patients [38]. These results support the concept that micro and macrovascular complications of diabetes share common pathogenic mechanisms beyond those related to the classical risk factors. The common pathogenic mechanisms between micro and macrovascular complications are uncertain but there is emerging evidence to suggest that endothelial dysfunction, platelet dysfunction, oxidative stress, inflammation, and advanced glycation end products are pathogenic factors for both micro and macrovascular complications in diabetic patients [39]. Although cost- effectiveness studies are still needed it is undeniable that DM2 could benefit from the identification of silent myocardial ischemia. In the DIAD study asymptomatic diabetic patients with moderate or large myocardial perfusion defects had a 6-fold greater cardiac risk than those with normal studies or small defects [34]. However, the meaning of a positive SPECT in the setting of true silent myocardial ischemia, in particular when perfusion deficits are of mild intensity, remains to be elucidated. Meanwhile, in terms of clinical practice it would be reasonable to be especially rigorous in achieving a tight control of risk factors and blood glucose in these patients. In the DIAD study, a more intensive treatment of cardiovascular risk factors was associated with the resolution of perfusion deficits [40]. In the study of Hernandez et al. [35], the probability of having myocardial perfusion defects in an asymptomatic diabetic patient with DR was 11.7 (IC95%: 3.7-37). Therefore, patients with DR are good target population for indicating SPECT. This information is important not only for the management of diabetic patients but also in terms of the economic burden. The current guidelines already identify the need for routine screening for DR. In addition to appropriate vision care, the detection of DR might now also warrant a fuller cardiac evaluation and closer follow-up to prevent the development of CAD.

### The goals of blood pressure in the patient with diabetes and hypertension

Hypertension is present in 20% to 60% of patients with type 2 diabetes, depending on age, ethnicity, obesity, and the presence of micro or macro albuminuria [41]. High BP substantially increases the risk of both macro and micro vascular complications, doubling the risk of all-cause mortality and stroke, tripling the risk of coronary heart disease and significantly hastening the progression of diabetic nephropathy, retinopathy, and neuropathy [41-43]. In these patients, a difference of 5 mmHg in either systolic blood pressure (SBP) or diastolic blood pressure (DBP) increases the risk of cardiovascular events or death by 20% to 30% [44]. In observational studies, people with both diabetes and hypertension have approximately twice the risk of cardiovascular disease as no diabetic people with hypertension, and are also at increased risk for diabetes specific complications, including retinopathy and nephropathy. In the UKPDS epidemiologic analysis, for each 10 mmHg decrease in mean SBP the estimated risk of any complication related to diabetes, deaths related to diabetes, MI, and micro vascular complications was reduced by 12%, 15%, 11%, and 13%, respectively [45]. These results are in keeping with the 15% risk reduction for cardiovascular death reported in the Seven Countries Study and to the estimates of the Multiple Risk Factor Intervention Trial (MRFIT) for diabetic patients [46,47]. Thus, BP lowering is a major priority in preventing cardiovascular and renal events in patients with DM2 and hypertension.

During many years the BP goals recommended in patients with diabetes were more aggressive than in patients without diabetes [48-53]. Moreover, it was proposed the maximum of “the lower the better” with no threshold [53]. However, the 2009 European Society of Hypertension guidelines proposed that lowering the blood pressure to less than 130/80 mmHg in patients at high risk for cardiovascular events was unsupported by prospective trial data, and that the systolic blood pressure should be lowered to less than 140 mmHg in these patients [54]. Moreover, this reappraisal of the European guidelines addressed the issue of the so- called J-curve and the clinical implications from this phenomenon, subject that has been reviewed recently [55-58]. The recommendations suggest lowering the SBP/DBP to values within the 130-139/80- 85 mmHg range in all hypertensive patients [54]. Several news clinical trials, including a retrospective analysis supported this recommendation. The International Verapamil SR- Trandolapril Study (INVEST) [59] in which the patients were divided into three groups depending on the achieved BP: (1) those who had not reached the control level (SBP >140 mmHg), (2) those who had reached the standard control level (SBP <140-130 mmHg) and (3) those on intensive BP control (SBP <130 mmHg). In patients with non-controlled BP, the risk of death, MI or stroke was as much as 50% higher compared to those with controlled BP (HR 1.46; p < 0.0001). Interestingly, it was observed an increased risk of death due to any cause - about 8% after 30 months and 5 years after the study [adjusted HR: 1.20 (p = 0.06) and 1.15 (p = 0.04), respectively] in patients with intensively controlled BP. Additional analyses revealed that this risk was caused by a higher incidence of death in patients with SBP below 115 mmHg [59]. The Action to Control Cardiovascular Risk In Diabetes - Blood Pressure Arm (ACCORD-BP) study [44] was designed to evaluate the impact of treatment aimed at intensive lowering of SBP to <120 mmHg (compared to standard therapy) on the incidence of CV events in 4,733 DM patients. The study enrolled high-risk DM patients: aged >40 and with coexisting CVD; or aged >55 with marked atherosclerosis, albuminuria, and LVH; or with at least two risk factors for CVD: dyslipidemia, arterial hypertension, smoking or obesity. After a year of treatment, the mean SBP was 119.3 mmHg in the group managed intensively and 133.5 mmHg in the group on standard therapy, while the mean DBP values were 64.4 and 70.5 mmHg, respectively. The primary endpoint, comprising nonfatal MI or stroke, or death due to CV causes, occurred in 445 patients (1.87% per year in the group on intensive treatment compared with 2.09% of those on standard therapy; p = 0.20). In addition, there were 294 deaths due to any cause (1.28% in the intensive therapy group vs. 1.19% in the standard treatment group; p =0.55) and 118 due to cardiovascular causes (0.52% vs. 0.49%, respectively; p = 0.74). The incidence of stroke was significantly higher in the group receiving standard treatment (0.53% vs. 0.32%; p = 0.01); a similar relationship was found for non-fatal stroke (0.30% vs. 0.47%; p = 0.03). It was concluded that intensive hypotensive therapy did not significantly reduce the incidence of primary endpoints or the majority of secondary endpoints; however, it was associated with a significant reduction in the total number of strokes (by 41%; HR 0.59; 95% CI, 0.39-0.89; p = 0.03) and nonfatal strokes (by 37%). In the intensive therapy group, the incidence of adverse complications of treatment (orthostatic hypotension, hyperkalemia, syncope, bradycardia, arrhythmia or renal function impairment) was significantly increased (3.3% vs. 1.3%). Thus, the ACCORD-BP study demonstrated the importance of defines the group of patients in whom significant BP reduction could be particularly dangerous and, on the other hand, those with a high risk of stroke who could benefit most from an intensive hypotensive therapy. Moreover, the ACCORD-BP study confirmed that lowering SBP to below 115 mmHg may be dangerous [44].

In the Irbesartan Diabetic Nephropathy Trial (IDNT) [60], similar results were observed where a DBP <85 mmHg was associated with an increase in all-cause mortality, a significant increase in MI, but a decreased risk for stroke. The Appropriate Blood Pressure Control in Diabetes-Normotension (ABCD-NT) trial [61,62], a level of SBP <130 mmHg was not associated with a benefit in the primary outcome (renal dysfunction) or any other CV outcome. The active group participants did benefit from a significant reduction in stroke. This trial randomized 470 subjects with type 2 diabetes (age: 58 years; baseline BP: 155/98 mmHg) to a DBP target of either 75 mmHg or 80 to 89 mmHg. Achieved BP was 132/78 and 138/86 mmHg in the intensive- and the less-intensive groups, respectively. No significant difference in any cardiovascular end points, rate of progression of renal disease, or retinopathy was reported, even if a difference in overall mortality was also evident (6% and 11% in the intensive and less-intensive groups, respectively).

A lack of benefits of lowering the SBP level <130 mmHg in patients with diabetes was also observed in the recent analysis of the ONTARGET trial [63]. In this study, an increased cardiovascular mortality was observed in the presence of SBP lower than 125 mmHg, compared with SBP less than 130 mmHg. These results are consistent with the olmesartan study, wherein a SBP lower than 120 mmHg showed a J-shaped increase of cardiovascular mortality in the olmesartan group, compared with the placebo group [64].

Vamos et al. [65] included a total of 126,092 adult patients (age >18 years) from the United Kingdom General Practice Research Database (UKGPRD) with a new diagnosis of type 2 diabetes. This study demonstrated not only the lack of benefits of lowering the SBP below 130 mmHg, but also the J-shaped relationship in DM patients. In patients with CVD, the tight control of SBP (<130 mmHg) and DBP (<80 mmHg) was not associated with improved survival.

The HOT trial included a prespecified subgroup analysis in 1,501 diabetic subjects (age: 61.5 years; baseline BP: 170/105 mmHg), randomized to three different DBP targets: 90, 85, and 80 mmHg [66]. Achieved BP was 144/85, 141/83, and 140/81 mmHg in the three target groups, respectively. A target of 80 mmHg significantly reduced both major cardiovascular event rates (11.9/1,000 person-years; RR 0.49; 95% CI, 0.29-0.81) and cardiovascular mortality (3.7/1,000 person-years; RR 0.33; 95% CI, 0.14-0.78) compared with a target of 90 mmHg (24.4/1,000 person-years and 11.1/1,000 person-years, respectively).

The results of the HOT study give place a very intensive discussion about the J-shaped existence [67-69]. As was discussed by Cooper-DeHoff et al. [57] and Garcia-Touze and Sowers [70] in recently reviews, one of the main concerns on intensive BP lowering is the belief that excessive reduction of BP values, particularly diastolic, may increase the risk of MI. In diabetic patients with hypertension, two post hoc observational analyses have recently raised the issue of a J-curve effect [71,72]. In the first study [71], 6,400 patients with diabetes, CAD, and age >50 years, from the original 22,576 participants of the INVEST trial, were divided into three different groups: tight control (SBP <130 mmHg), usual control (from 130 mmHg to <140 mmHg), and uncontrolled (140 mmHg or more). Primary outcome was the first occurrence of all-cause death, nonfatal MI, or nonfatal stroke. During 16,893 patient-years of follow-up, 286 patients (12.7%) with tight BP control, 249 (12.6%) with usual control, and 431 (19.8%) with uncontrolled SBP experienced a primary outcome event. Although usual BP control allowed a significant reduction in the cardiovascular event rate, as compared with the uncontrolled group (12.6% vs. 19.8%; adjusted hazard ratio [HR], 1.46; 95% CI, 1.25-1.71; P < 0.001), little difference existed between those with usual control and those with tight control (12.6% vs. 12.7%; adjusted HR, 1.11; 95% CI, 0.93-1.32; P = 0.24). In addition, all-cause mortality rate showed a no statistically significant trend toward a higher risk in the tight-control group, as compared with the usual control group (11% vs. 10.2%; P = 0.06), that became statistically significant when the extended follow-up was included (22.8 vs. 21.8%; P = 0.04). A major limitation of the latter finding was the fact that no BP data were collected during the extended follow-up [73].

In the second study, a post hoc observational analysis on the diabetic patients of the UKPDS [72] investigators randomly assigned patients with diabetes to either ‘tight BP control’ (<150/85 mmHg) or ‘less-tight BP control’ (<180/105 mmHg). Patients in the <150/85 mmHg group had a mean baseline BP of 159/94 mmHg and achieved a mean BP of 144/82 mmHg, whereas those in the <180/105 mmHg group had a mean baseline BP of 160/94 mmHg and achieved a mean BP of 154/87 mmHg after more than 8 years of follow- up. Compared with the less-tight-control group, those in the tight-control group had a significant 44% reduction in risk of stroke (P = 0.013), a 32% reduction in risk of diabetes- related death (P = 0.019), and a 24% reduction in risk of diabetes-related end points (P = 0.0046). When the UKPDS investigators performed an additional 10-year follow-up of the patients [73], which included in-person visits and questionnaires but no attempt to intervene on BP, the benefits observed in the tight-control group at the first 8-year follow-up were no longer present. Over the entire 20-year follow-up period, no difference in the rate of any diabetes-related end points, MI, microvascular disease, or all-cause mortality was observed between the tight-control and less-tight-control groups [73]. The Table 1 summarizes the main results of the clinical trials about discussed.

**Table 1:** Studies addressing the definition of the goal of blood pressure control in patients with diabetes and hypertension

**Study**	**Ref**	**Number of subjects**	**Design**	**BP (mmHg) target by groups**	**BP (mmHg) achieved**	**Main results**
INVEST	58	6,400	Observational subgroup analysis	Group 1. SBP<140	**Group 1. 159/86**	Group 1 have 50% higher risk of death, MI, or stroke (P< 0.0001). Group 3 in relation to group 2 have an increase of 8% of CVD after 5 years of study (p<0.04).
				Group 2. SBP<140-130	**Group 2. 149/85**	
						Higher incidence of death in patients with SBP<115.
				Group 3. SBP<130	**Group 3. 144/85**	
ACCORD-BP	43	4,733	Randomized clinical trial (RCT)	Group 1. Intensive SBP<120	Group 1. 119.3/64.4	No differences in the primary end point (MI, stroke and CV death) or in death due to any cause. Higher incidence of stroke (p= 0.01) or non-fatal stroke (p=0.03). Increase of adverse events in group 1.
				Group 2. Standard SBP < 140	Group 2. 133.5/70.5	
IDN-T	59	1,590	Post hoc analysis	≤135/85	30% reach the SBP goal and 81% the DBP goal	Progressively lower achieved SBP to 120 predicted a decrease in CV mortality and CHF but not MI. A SBP <120 was associated with increased CV deaths and CHF events. DBP< 85 was associated with increase of all-cause mortality, MI mortality but decrease risk of stroke.
ABCD-NT	60,61	470	RCT	Group 1. DBP<75-79	132/78	No differences in any CV events, or progression of renal disease, nor retinopathy.
				Group 2. DBP 80-89	138/86	
ONTARGET	62	9,603	Post hoc analysis	Group 1. SBP 95-130	125.8 SD 12.0	Increased CV mortality with SBP < 125 in relation with SBP < 130.
				Group 2. SBP 131-142	132.4 SD 11.2	
				Group 3. SBP 143-154	137.7 SD 11.5	
				Group 4. SBP 155-200	144.3 SD 12.6	
ROADMAP	63	4,447	RCT	Group 1. SBP<130	80% achieved the target	SBP<120 showed a J-shaped increase of CV mortality.
				Group 2. DBP<80		
UKGPRD	64	126,092	Retrospective study	Group 1. <130/<80	Achieved target	J-shaped relationship in patients with SBP<130 In patients with CVD SBP <130 and DBP< 80 was not associated with improved survival. BP <110/75 increase the risk of CV mortality.
		12,379 with CVD		Group 2. 130-139/80-<85	Group 1. SBP18.1%	
				Group 3. ≥140/≥85	DBP 35.7%	
					Group 2. SBP 19.9%	
					DBP 27.7%	
					Group 3. SBP 61%	
					DBP 36.6%	
HOT	65	1,501	RCT	Group 1. DBP<90	Group 1. 144/85	DBP<80 showed a significantly reduction in CV events and CV mortality.
				Group 2. DBP<85	Group2. 141/83	
				Group 3. DBP<80	Group 3. 140/81	
UKPDS	71	4,801	Post hoc observational analysis	Group 1. <150/85	Group 1. 144/82	Group 1 had a significant 44% reduction of stroke, 32% of diabetes related death, 24% diabetes related end patients.
				Group 2. <180/105	Group 2. 154/87	

BP= blood pressure, SBP=systolic BP, DBP= diastolic BP, MI=myocardial infarction, CVD= cardiovascular diseases CV=cardiovascular, CHF= congestive heart failure.

We refer the above-cited reviews [56,57,70] for the explanation of the pathophysiologic interpretation of the J-curve that lies in the mechanisms of coronary artery perfusion, which depends on the pressure gradient between the coronary arteries and the left ventricle during the diastolic phase of the cardiac cycle. However, the concept of a J-curve has been challenged by several observational studies and randomized controlled trials, in which the J-curve effect was not clearly evident. In addition, other explanations about the finding of a J-curve have been proposed; in particular, reverse causality views coexisting in chronic diseases or poor health conditions as a cause for concomitant low DBP, which could lead to a spurious association with increased morbidity and mortality.

Chronic kidney disease (CKD), defined by a reduced glomerular filtration rate (GFR) is common in subjects with DM2 and hypertension [74,75]. Nowadays CKD is affecting 10-15% of the adult general population and it is associated with an increased risk of CVD [76]. Guidelines recommended lower BP targets in this population than in people without CKD [77]. Moreover, it has been suggested that the inhibitors of the RAAS have particular benefits for the prevention of renal complication [78]. A recent meta-analysis of randomized controlled trials [79] that included 25 trials and 152,290 individuals showed that BP lowering regimens in relation to placebo reduced the risk of mayor CV events in individuals with and without reduced GFR with no evidence for any difference in effect. The results were similar irrespective of whether BP was reduced by regimens based on different antihypertensive drugs. This meta-analysis provides clean evidence that a broad range of different BP lowering regiments provide protection against CVD in patient with CKD. However, the results of the ONTARGET trial [21] and of the VA NEPHRON study [80] have clearly demonstrated that the combination of an ACEI plus an ARB not is recommend because of the increased risk of adverse events among patients with diabetic nephropathy. Moreover, as revised by Wu et al. [22], two large scale placebo controlled trials for diabetic nephropathy using the ARBs Irbesartan and Olmesartan [81,82] have shown a higher rate of CV death among patients randomized to the ARB group. On the other hand, the ONTARGET trial [21,83] using the ARB Telmisartan and the ACEI ramipril, showed equivalent cardioprotective effects of both RAAS blockers in patients with high risk of CVD or DM2. Nevertheless, participants in this trial were not randomized based on the presence of DM2 or the severity of nephropathy. The meta-analysis of Wu [22] concludes that since there is no evidence to show a better protective effect for ARBs compared with ACEI, the use of ACEI is recommended when cost is a concern.

## Conclusions

The importance of BP as a modifiable risk factor for CVD, especially in patients with diabetes is well recognized, and many effective and inexpensive BP-lowering treatments are commonly available. The recent Latin American (1) and European (2) guidelines, published this year has recommended as a goal for the blood pressure control in patients with diabetes type 2 a value similar or inferior to 140/90 mmHg. Therefore, BP control and prevention of related morbidity and mortality is clearly achievable. However, the awareness, treatment and control of hypertension are low worldwide. The big challenge now is avoid the therapeutic inertia (leaving diabetic patients with BP values of 140/90 mmHg or higher) at all costs, as this would lead to an unacceptable toll in terms of human lives, suffering, and socioeconomic costs. The health team and the subjects affected of hypertension and diabetes must to participate actively in the challenge to pass from the guidelines recommendations to the clinical practice and public health programs, to improve the percentage of control of high BP. Moreover, the development of research aimed to evaluate new approaches to effectively diagnostic, treated and control high BP is a priority [84].

## Abbreviations

ABCD-NT: Appropriate blood pressure control in diabetes-normotension trial; ACCORD-BP: Action to control cardiovascular risk in diabetes - blood pressure arm; BP: Blood pressure; BPLTTC: Blood pressure lowering treatment trialists collaboration; CAD: Coronary artery disease; DBP: Diastolic blood pressure; CVD: Cardio vascular diseases; DALY: Disability adjusted life years; DIAD: Detection of ischaemia in asymptomatic diabetics study; DM2: Diabetes type 2; DR: Diabetes retinopathy; HIC: High income countries; HOT: Hypertension optimal treatment trial; IDNT: Irbesartan diabetic nephropaty trial; IHD: Ischemic heart disease; INVEST: International verapamil SR-trandolapril study; LIC: Low income countries; LMIC: Low middle income countries; MI: Myocardial infarction; MIC: Middle income countries; MRFIT: Multiple risk factor intervention trial; ONTARGET: Ongoing telmisartan alone and in combination with ramipril global endpoint trial; PURE: Prospective urban rural epidemiology; RASS: Renin-angiotensin-aldosterone system; SBP: Systolic blood pressure; UKPDS: United Kingdom Prospective Diabetes Study; UMIC: Upper middle income countries.

## Competing interests

The authors declare that they have no competing interests.

## Authors’ contributions

PL-J and IR-A conceive and design the manuscript JL-L and CL-L participate in acquisition of data. All authors have been involved in drafting the manuscript and have given final approval of the version to be published.
